# Selection criteria for laboratory robotic application personnel

**DOI:** 10.1155/S1463924692000129

**Published:** 1992

**Authors:** Peter W. Rulon

**Affiliations:** Advanced Technology and Validation Section Quality Assurance Department Norwich Eaton Pharmaceuticals, Inc. Norwich New York 13815 USA

## Abstract

Norwich Eaton Pharmaceutical recognized the benefits of using automation systems in the laboratory over seven years ago and created a robotic development area within the analytical method development group. They now have eight complete robotic systems and a large number of semi-automated systems in routine operation.

This level of activity has provided many challenges for the automation group. The success of this group has been very dependent on the talents of people working these assignments. You can have the best equipment and the vendor's promises of success, but it is the people who understand the products and the requirements that get systems on line.

Assembling an effective robotics organization requires prework on the part of management. There must be a clear vision of the specific types of activities the group will perform. This vision can be used to establish a skills profile for the members of the team. It appears that at least four people are required to provide the variety of skills and keep the group going.

Each member's personality is an important component of establishing a new team. In robotics, one of the most critical talents is the ability to work on long term projects that constantly present new challenges. The group members need to balance consistency of purpose with the ability to creatively solve a variety of problems. The group will not be effective in delivering new technologies unless they have the talent to train the novice in a highly technical environment.

People who are successful in automation development are unique. They should have the ability to work comfortably in a logic-based environment, to become very creative on demand, and to communicate highly technical information effectively. People do not usually possess all these skills, providing their manager with challenging coaching opportunities.


Journal of Automatic Chemistry, Vol. 14, No. 2 (March–April 1992), pp. 51–53

### Selection criteria for laboratory robotic application personnel

**Peter W. Rulon**

*Advanced Technology and Validation Section, Quality Assurance Department, Norwich Eaton Pharmaceuticals, Inc., Norwich, New York 13815, USA*

*Norwich Eaton Pharmaceutical recognized the benefits of using automation systems in the laboratory over seven years ago and created a robotic development area within the analytical method development group. They now have eight complete robotic systems and a large number of semi-automated systems in routine operation.*

*This level of activity has provided many challenges for the automation group. The success of this group has been very dependent on the talents of people working these assignments. You can have the best equipment and the vendor's promises of success, but it is the people who understand the products and the requirements that get systems on line.*

*Assembling an effective robotics organization requires prework on the part of management. There must be a clear vision of the specific types of activities the group will perform. This vision can be used to establish a skills profile for the members of the team. It appears that at least four people are required to provide the variety of skills and keep the group going.*

*Each member's personality is an important component of establishing a new team. In robotics, one of the most critical talents is the ability to work on long term projects that constantly present new challenges. The group members need to balance consistency of purpose with the ability to creatively solve a variety of problems. The group will not be effective in delivering new technologies unless they have the talent to train the novice in a highly technical environment.*

*People who are successful in automation development are unique. They should have the ability to work comfortably in a logic-based environment, to become very creative on demand, and to communicate highly technical information effectively. People do not usually possess all these skills, providing their manager with challenging coaching opportunities.*

### Introduction

Norwich Eaton Pharmaceuticals, Inc. have eight robots in routine use, as well as a large number of semi-automated systems. This could not have happened without the right people and this paper describes how to identify and develop the right people to assure long term success.

Automated procedures have had a substantial impact on the success of the analytical laboratories at Norwich Eaton. The savings from reduced labour costs exceeds $1m. An equally important benefit of using robots is the freedom that laboratory staff get from performing redundant analyses. This allows them to do more enjoyable and creative things with their minds.

Bringing automation to the laboratory is not an easy task. There is a lot to learn by studying why one organization succeeds and another does not. These show that the automation development group must be properly staffed, programmes for technology transfer must be designed, and it is essential to provide on-site training and support systems.

Norwich Eaton has a group of 10 people in its Advanced Technology and Validation Section (ATV). This group reports into the QA organization. Their mission covers a variety of analytical responsibilities, including the development of automation systems. On average there are three full-time people working on automation.

Automation development provides many challenges. Success or failure depends on the number and talents of the people working on projects. ATV can have the best equipment and the vendor's promises of success, but experience has shown that it is the people who understand the products and the requirements that get our sytsem on-line.

### Preplan

When a business decides that it needs automation, it must start with a plan and that plan should include specific details on specific requirements for the personnel needed to staff that function. It is impossible to staff a group, until it is known exactly what skills that group needs. Once the list of required skills is established, it is useful to separate them into two categories of 'must have' skills and 'nice to have' skills. It is also useful to look at what skills are readily trainable and what natural talents are required to be effective. Once the skills are outlined and well understood, developing a staffing plan becomes straightforward.

This type of skills analysis is also a useful tool to enhance an established automation group. In this case, their required skills list is matched against each individual's personal skill assessment. In this way, skill gaps – or training opportunities – are readily identified. The data can then be used to formulate plans to address areas of weakness or plan for the better use of strengths.

To describe the concept of skill analysis a typical robotic project is reviewed focusing on what skills are needed.

### Staffing for success

Most automated development projects have several common phases. Each phase requires some unique skills. Finding someone with all the required skills is a challenge, if not an impossibility. Fortunately many of the skills are readily trainable.

© Zymark Corporation 1992

*Phase I – The salesman*

Opportunities for automation projects comes from a variety of sources. The potential user site many respond to this idea with overwhelming excitement or with a reply such as: 'You're not putting THAT thing in my lab'. Before starting, a shared expectations contract needs to be negotiated. Staff need the skill to sell the benefits of the proposal, provide a balanced explanation of any limitations, negotiate timing and validate that the proposed system will meet customer needs. Here is where interpersonal skills, especially in negotiation and persuasion, are important.

*Phase II – The programmer—engineer*

Once the project goal is defined, the first step in most automation projects is writing computer programmes to perform the analysis. The complexity of this task varies greatly depending in part, on the complexity of the task. The challenge becomes more complex because most systems have their own software with their own set of commands, formats and structure. A dry run of the system is made after the first draft of the code is written and our experience is that things never run as planned. In an iterative process the code must be edited, tested and edited again until the system successfully passes the dry run test.

The ability to be a effective programmer comes from a set of natural talents. Good programmers are capable of focusing on a complex set of commands and understanding how they interact. During the initial testing phase it is quickly apparent that most systems have components that do not work as desired – they need to be modified to perform the task intended. This step requires the skills of a programmer and engineer ('tinkerer'). This phase also needs creativity/innovation.

System trials usually point out opportunities to improve the hardware. It is common for subsystems to face reliability problems or even worse; not perform their task at all. Major system failures are usually referred back to the vendor, but small modifications are typically performed by the development staff. At this point, it is necessary to use the talents of other disciplines.

*Phase III – Chemist*

Once the prototype system has passed the dry run test, the next step is to try a test run on the actual analysis. The role of the programmer/engineer never ends, but completion of this phase usually requires more of the skills of an analytical chemist. The first trial run of a 'real' assay never works properly. Often the problem lies in the downsizing of the analysis to fit the automation system.

This phase requires a good experimentalist. The system developer should have an understanding of the chemistry of the assay. This understanding will lead to the required experimental studies to resolve the problems. The chemist's understanding of bias, response curves, solubility, reaction chemistry and solids errors, all come into play in solving method problems. Once all method problems are resolved, the analytical chemist's talents in method validation are critical. This is typically an interative process using all the skills outlined in phase II and III to achieve a working solution.

*Phase IV – Teacher/trainer*

The next step is to transfer this properly operating automated system from the development laboratories to the user site. Setting up the equipment at the user site can be easy if the entire unit is transferred. Modifications of existing equipment at user's sites is more complex and requires much more talent. Experimentally validating system operation at a new site is simple to design and interpret for an experienced analytical method development chemist.

The hard part of technology transfer is training the new operators to run, maintain and trouble-shoot the system. It is difficult for the developer to remember how long it took for them to learn the system. The magnitude of time required for cross training is often underestimated.

The basic talents required for the training/transfer phase of the transfer are typically not found in someone trained as a programmer/engineer/chemist. This usually presents a major challenge to their interpersonal skills. They need to use their skills to encourage ownership and confidence in the new system. Teaching adults new technologies is difficult for many people because they do not realize that the way they were taught at school does not work well when training an adult. There are specific training skills that have been shown to be effective for adults. All this requires a basic sensitivity to the needs of the customer. That sensitivity to another person's needs is a difficult skill to teach.

### Manager's challenge

The skills analysis that the author has described suggests that a group must be staffed with people who can be salesman, negotiators, programmers, engineers, chemists, and teachers with good listening skills and communicate well in writing and speech. It is unlikely that a recruiting programme will find such a person. So the next step is to decide what the 'must have skills' are. 'Must have' natural talents include a basic understanding of analytical chemistry; ability to work on long-term detailed project activities, and reasonable interpersonal skills. Much of the rest can be trained

### Staffing levels

Experience at Norwich suggests that a minimum of three, and preferably four people need to be working in automation at any one time. This minimum number of people appears to provide the necessary critical mass for long-term continuity. Long lead times are required to bring in a new member of the group. A group size of at least four assures that the loss of one staff member to another assignment does not fully shut down the operation. There is also significant synergy from a staff of at least four people – others can provide assistance to help each other when they face their talent gaps.

### User laboratory staffing

The job is not done once the robotic system has been finished. It is just as important, or even more important, to plan the staffing skills at the automation user site. An important role for the development staff is to identify the required skills at the user site. The developers have the shared responsibility to ensure that the user site is prepared for new technology.

There are at least two levels of users and the skills should be matched to the expectations. Every site will have a set of operators and should also have at least one site expert. The site expert should generally have a scaled-down version of the developer skills. That person should be proficient in details of the basic code, chemistry of the analysis, training others, understanding how to calibrate and maintain the system. The expert should be able to assure continuing operation with little contact with the development laboratory. In time, expert's knowledge of the system will exceed that of the developers. The skill to diagnose and maintain mechanical systems is critical in this role. This position should be filled by someone who can assemble a new bicycle from the box in less than 30 minutes. This person is critical to success, so a back-up training programme is important.

The unique skills demands for operators is much more limited. Most laboratory staff can be trained in the basic system operation and maintenance. It is important to provide training in diagnosing and resolving common problems: the more operators are taught, the more likely the project will be a success.

### The manager

No group can be successful in a vacuum. Many automation projects are long term and demanding. The effective manager should set an environment that minimizes distractions, supports creativity, encourages communications, garnishes the support of upper management. In general these are all skills expected of a good manager; however, the management challenge for an automation group is *much greater.*

The extra demand on the manager come partly from the extra demands on the staff to use a variety of skills. The manager needs to help each individual develop formal training plans and follow up on their completion. Projects are typically long term and require the manager to work with staff on a regular basis to assure alignment with project goals and to show personal interest in success. The manager should make sure that formal plans and responsibility assignments are given for the technology process. Another role for the manager is to encourage creativity and risk taking in the group. Managing an automation group requires established interpersonal and leadership skills; this is not a job for a new manager.

### Training tools

It is easy to find lists of training programmes that claim to address all types of needs. Selecting the most effective programme comes from experience (often someone else's) and understanding the learning style of the individual. Training can range from a formal schooling programme to simply assigning a person to work with an expert.

Many of the major vendors provide good schools on system programming and repair. They can rapidly bring a new person up to speed in this area. There is also a variety of interpersonal skills training programmes offered by many vendors, and usually by in-house staff. The best programmes are those which offer opportunities to practise skills. Basic analytical chemistry skills are critical; creative thinking skills can be taught through several programmes. With proper planning most skill gaps can be minimized and there are many programmes that will help strengthen exisiting skills.

### Other development tools

An experiment was carried out at Norwich Eaton that provided real value in understanding how we work. All members of the author's section took the Myer's-Briggs Type Indicator test [1]. Most of the group's identified personality type was INTJ. The data points suggests that the group lacks some diversity, and, indeed, the similarities do present some problems in group dynamics. The fact that most of the group tested introverts (the I in INTJ) means there is a coaching challenge when the group needs to communicate with other departments.

There are also learning styles tests that can provide an individual with some insight on what is the most effective training for them. People learn in a variety of ways. Some learn better from instruction and others learn better from experience.

### People are the key to success

Even with a room full of state-of-the-art automation equipment, the best fiscal plans, the right facilities, commitment from management, without the right people an automation project will not be a success.

It is vital to plan personnel issues before the start of a project. Skill analysis can help with the right staffing.

### Note

1. Myers-Briggs Type Indicator is a registered trademark of Consulting Philologists Press Inc., Palo Alto, California, USA.

